# The Burden and Genetic Diversity of *Mycobacterium bovis* Infection in Humans in the Middle East and North Africa: A Systematic Review and Meta‐Analysis

**DOI:** 10.1155/tbed/5876735

**Published:** 2026-10-06

**Authors:** Tsegaye Shamebo, Aboma Zewude, Temesgen Mohammed, Nabeeha Abdelgaleel Hassan, Robert Barigye, Markos Tibbo, Ali Artaman, Gobena Ameni

**Affiliations:** ^1^ Department of Health Sciences College of Natural and Health Sciences Zayed University P.O. Box 144534, Academic City Dubai UAE zu.ac.ae; ^2^ Department of Veterinary Medicine College of Agriculture and Veterinary Medicine United Arab Emirates University P.O. Box 15551 Al Ain City Abu Dhabi UAE uaeu.ac.ae; ^3^ Environmental and Occupational Health Institute of Public Health College of Medicine and Health Sciences United Arab Emirates University PO Box 1555 Al Ain City Abu Dhabi UAE uaeu.ac.ae; ^4^ Subregional Office for the Gulf-Cooperation Council States and Yemen Food and Agriculture Organization of the United Nations P.O. Box 62072 Abu Dhabi City Abu Dhabi Emirate UAE fao.org

**Keywords:** clonal complex, genetic diversity, MENA region, *Mycobacterium bovis*, prevalence, zoonotic tuberculosis

## Abstract

Zoonotic tuberculosis (zTB) includes human tuberculosis caused by animal‐associated *Mycobacterium* species, *Mycobacterium bovis* (*M. bovis*) being the major cause of zTB. This systematic review and meta‐analysis was conducted to estimate the pooled burden of human *M. bovis* infection and describe the genetic diversity of *M. bovis* isolated from humans in the Middle East and North Africa (MENA) region. A literature search was conducted in PubMed/MEDLINE, Scopus, Web of Science, Embase, and Google Scholar, followed by manual screening of additional references. The literature used for this paper were published between 2000 and 2025. A random‐effects meta‐analysis was used to estimate the pooled prevalence of human *M. bovis* infection. Heterogeneity was assessed using the *I* ^2^ statistic, while sensitivity analysis and Egger’s regression test were used to assess the robustness of the findings and potential small‐study effects, respectively. Totally, 27 studies from 10 MENA countries involving 6224 participants and 154 *M. bovis*–infected cases were included. The pooled prevalence of human *M. bovis* infection was 3% (95% CI: 2%–4%), with substantial heterogeneity (*I* ^2^ = 73.9%). The pooled prevalence was 4% in North Africa and 3% in the Middle East (*p* = 0.408). The individual study did not have an effect on the pooled prevalence as the leave‐one‐out sensitivity analysis did not change the pooled prevalence. The funnel plot showed some visual asymmetry, while Egger’s regression test did not provide statistical evidence of small‐study effects (*p* = 0.852). The *M. bovis* isolated from humans in MENA region were classified under European 1 (Eu1) and European 2 (Eu2) clonal complexes. In conclusion, the review highlighted the significance of *M. bovis* infection in humans in the MENA region, although there is a shortage of epidemiological data from more than 50% of the countries of the region. The isolation of Eu1 and Eu2 *M. bovis* clonal complexes from human *M. bovis* isolates suggested the importation was infected from Europe, which further transmitted the strains to humans. Strengthening of a One Health approach–based surveillance and molecular epidemiological studies in all MENA countries would contribute to the control of *M. bovis* in animals and humans in the region.

## 1. Introduction

Zoonotic tuberculosis (zTB) refers broadly to tuberculosis caused by animal‐associated members of the *Mycobacterium tuberculosis* complex (MTBC). Among these organisms, *Mycobacterium bovis* (*M. bovis*) is an important cause of tuberculosis in cattle and a recognized zoonotic pathogen in humans [1, 2]. The present review specifically focuses on human *M. bovis* infection and does not estimate the burden caused by other zoonotic MTBC species, such as *M. caprae* or *M. pinnipedii*. As such, zTB caused by *M. bovis* represents a critical interface between human and animal health, underscoring the importance of integrated One Health approaches for effective disease surveillance and control [3].

Human infection with *M. bovis* typically occurs through occupational exposure to infected livestock, inhalation of contaminated aerosols in farm or abattoir environments, or the consumption of unpasteurized milk and dairy products derived from infected animals [4, 5]. Consequently, livestock farm workers, veterinarians, abattoir workers, dairy handlers, and pastoralist communities are at elevated risk of exposure. In settings where bovine tuberculosis (bTB) control is inadequate and food safety measures are limited, zTB caused by *M. bovis* may account for a substantial proportion of total TB cases [6, 7]. The public health significance of *M. bovis* infection is further heightened by the intrinsic resistance of the organism to pyrazinamide, a key first‐line anti‐TB drug, complicating therapeutic management when species identification is not performed [8].

Human *M. bovis* infection is likely underdetected in many settings because routine tuberculosis diagnostic approaches frequently identify MTBC without routinely distinguishing *M. bovis* from *M. tuberculosis*. This diagnostic limitation can result in *M. bovis* infections being classified as tuberculosis without species‐level attribution [2, 7, 8]. Consequently, the epidemiology of human *M. bovis* infection remains incompletely characterized, particularly in settings with limited access to culture confirmation and molecular speciation.

The Middle East and North Africa (MENA) region constitutes a distinctive epidemiological setting for human *M. bovis* infection, influenced by diverse livestock production systems, long‐established pastoral practices, and a strong dependence on animal‐derived food products [4, 5]. Across multiple countries, including Egypt, Iran, Morocco, Algeria, Sudan, Tunisia, and Turkey, livestock such as cattle, goats, sheep, and camels play a central role in sustaining rural livelihoods and providing essential sources of nutrition and household income [7]. Close and sustained interaction between humans and animals in these environments increases the risk of cross‐species transmission of *M*. *bovis*, the causative agent of bTB [3, 4]. In addition, structural and systemic challenges, including informal livestock markets, unregulated transboundary animal movement, and limited implementation of veterinary public health measures, contribute to the persistence and spread of bTB within animal populations [5, 9]. These interconnected factors collectively sustain the transmission cycle at the human–animal interface and complicate efforts to control zTB caused by *M. bovis* in the region [7–9]. Epidemiological studies conducted across several MENA countries have documented the presence of *M. bovis* in both animal reservoirs and human TB cases; however, the reported prevalence varies widely, while the agent remains incompletely characterized. This fragmented and largely localized evidence, often based on small sample sizes, limits accurate estimation of the true regional burden of *M. bovis* infection and constrains generalizability [8]. Although the meta‐analytical approaches have been applied to synthesize these findings, they are assumed to provide more generalizable estimates than individual studies.

Assessing the burden of *M. bovis* infection requires a clear understanding of the molecular epidemiology of *M. bovis* in the MENA region, as it enables the identification of transmission routes and tracking of disease spread. To date, four *M. bovis* clonal complexes, including African 1 (Af 1), Af 2, European 1 (Eu1), and European 2 (Eu2), have been reported in the MENA region. These clonal complexes exhibit distinct geographic distributions, reflecting historical dissemination driven by livestock movement and trade [10, 11].

Although zTB may be caused by several zoonotic MTBC species, the present review was restricted to human *M. bovis* infection. Therefore, the prevalence estimates generated in this study should be interpreted as estimates of *M. bovis*–associated infection among the populations studied and not as estimates of the total burden of zTB caused by all zoonotic *Mycobacterium* species. Therefore, this systematic review and meta‐analysis aimed to estimate the pooled prevalence of human *M. bovis* infection in the MENA region and synthesize the available evidence on the molecular characteristics and distribution of *M. bovis* clonal complexes in humans.

## 2. Methods

### 2.1. Study Design

This systematic review and meta‐analysis was conducted to assess the burden of human *M. bovis* infection in the MENA region and to synthesize available evidence on the molecular characteristics of human *M. bovis* isolates. The review followed the Preferred Reporting Items for Systematic Reviews and Meta‐Analyses (PRISMA 2020) guidelines for study identification, screening, eligibility assessment, data extraction, and reporting [12]. The review protocol was prospectively registered in PROSPERO (CRD420261359603).

### 2.2. Literature Search Strategy

A systematic literature search was conducted to identify studies reporting human *M. bovis* infection in the MENA region. PubMed/MEDLINE, Scopus, Web of Science, Embase, and Google Scholar were searched from database inception to 15 February 2026. The search strategy combined controlled vocabulary and free‐text terms related to *M. bovis*, bTB, zTB, human tuberculosis, molecular epidemiology, and relevant diagnostic and genotyping methods, together with the names of MENA countries. Keywords included “zoonotic tuberculosis,” “bovine tuberculosis,” “*Mycobacterium bovis*,” “clonal complex,” “spoligotyping,” “MIRU‐VNTR,” “molecular epidemiology,” “risk groups,” “occupational exposure,” “Middle East,” and “North Africa.” Boolean operators “AND” and “OR” were used to combine search terms.

In addition to electronic database searching, the reference lists of included studies and relevant reviews were manually screened to identify potentially eligible publications. A total of 68 additional records were identified through manual reference–list searching and were incorporated into the PRISMA flow diagram. Only publications available in English were included.

### 2.3. Eligibility Criteria

Studies were eligible if they investigated human *M. bovis* infection or disease in populations residing in MENA countries. Eligible observational designs included cross‐sectional, cohort, and case–control studies, as well as surveillance studies containing relevant human data. Studies reporting prevalence, incidence, or molecular characterization of human *M. bovis* infection, including clonal complexes or genotypes, were considered eligible.

Studies reporting only other zoonotic MTBC species, including *M. caprae* and *M. pinnipedii*, were excluded because they were outside the predefined scope of the review. Studies involving only animal populations without human data were also excluded from the human prevalence analysis. Reviews, editorials, commentaries, conference abstracts without sufficient data, and case reports without relevant epidemiological information were excluded. Studies published before January 1, 2000, or after December 31, 2025, were excluded.

### 2.4. Study Selection Process

Records identified through database searches and manual screening of reference lists were combined, and the number retrieved from each source was documented in the PRISMA flow diagram (Figure 1). All records were imported into Zotero for duplicate removal.

**Figure 1:**
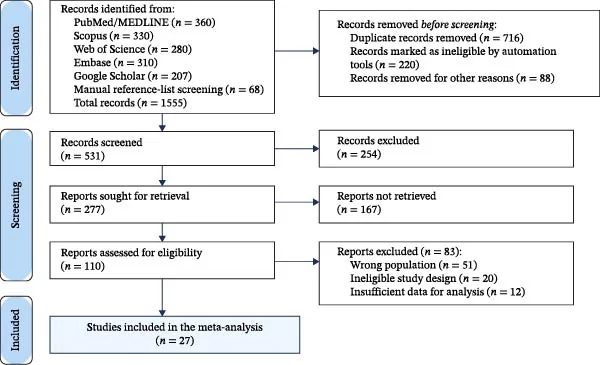
PRISMA flow diagram of the study selection process for the systematic review and meta‐analysis of human *M. bovis* infection in the MENA region. The diagram presents records identified through electronic databases and manual reference‐list searching, screening, full‐text assessment, and final inclusion.

Study selection was conducted in two stages. First, titles and abstracts were independently screened by two reviewers to identify potentially eligible studies. Second, the full texts of relevant articles were assessed against the predefined inclusion criteria. Disagreements between reviewers were resolved through discussion and consensus. The selection process was documented in accordance with PRISMA guidelines, including the numbers of records identified, screened, excluded, and ultimately included in the review.

### 2.5. Data Extraction and Variables

Data extraction was performed using a standardized and pretested data extraction form to ensure consistency and accuracy. The extracted variables included bibliographic details (author and year), country, study design, sample size, and characteristics of the study population.

Extracted variables included author, publication year, country, study design, study population, sample size, number of *M. bovis*–positive participants, diagnostic method, and molecular characterization results. Where available, information on spoligotype patterns, MIRU‐VNTR profiles, whole‐genome sequencing, and *M. bovis* clonal complexes was also extracted. Information concerning occupational exposure and risk groups was recorded where reported; however, these variables were not consistently available or sufficiently comparable across studies to support a reliable quantitative subgroup analysis.

### 2.6. Quality Assessment and Risk of Bias

The methodological quality and risk of bias of the included studies were assessed using an adapted Newcastle–Ottawa Scale (ANOS) (Supporting Information 1). The assessment considered study design, sampling methodology, population representativeness, diagnostic methods, outcome ascertainment, and completeness of reporting. Study design for each included study is presented in Table 1.

**Table 1:** Characteristics of studies included in the systematic review and meta‐analysis of human *M. bovis* infection in the MENA region.

Number	Author	Country	Population	Study design	Event	Total	Clonal complex	Genotyping method
1	Tazerart et al. [13]	Algeria	TB patients	Molecular epidemiological study	4	98	—	WGS
2	Damene et al. [14]	Algeria	TB patients	Molecular epidemiological study	7	115	European 2	Spoligotyping; PhyloSNP
3	Cooksey et al. [15]	Egypt	TB patients	Molecular epidemiological study	1	67	—	IS6110‐RFLP; spoligotyping
4	Abbadi et al. [16]	Egypt	TB patients	Molecular epidemiological study	1	45	—	IS6110‐RFLP; spoligotyping
5	Lobna and Nasr [17]	Egypt	Farm workers	Cross‐sectional molecular study	1	25	—	—
6	Abdelsadek et al. [18]	Egypt	Farm workers	Cross‐sectional molecular study	8	149	—	—
7	Asgharzadeh et al. [19]	Iran	TB patients	Molecular epidemiological study	15	165	—	IS6110‐RFLP; MIRU‐VNTR; ETR‐VNTR
8	Al‐Thwani and Al‐Mashhadani [20]	Iraq	TB patients	Molecular epidemiological study	2	186	—	—
9	Barak [21]	Iraq	Farm workers and veterinary doctors	Cross‐sectional molecular study	2	25	—	—
10	Bedrossian et al. [22]	Lebanon	Farm workers and veterinary doctors	Cross‐sectional molecular study	2	60	—	Spoligotyping
11	Al‐Hajoj et al. [23]	Saudi Arabia	TB patients	Molecular epidemiological study	13	1505	—	Spoligotyping; MIRU‐VNTR
12	Bayraktar et al. [24]	Turkey	TB patients	Molecular epidemiological study	8	188	—	—
13	Ağaçayak et al. [25]	Turkey	TB patients	Molecular epidemiological study	8	60	—	—
14	Öztürk et al. [26]	Turkey	TB patients	Molecular epidemiological study	3	220	—	GenoType MTBC
15	Smaoui et al. [27]	Tunisia	TB patients	Molecular epidemiological study	4	181	—	—
16	Soliman et al. [28]	Egypt	TB patients	Molecular epidemiological study	5	112	European 1	Whole‐genome sequencing
17	Soleimanpour et al. [29]	Iran	TB patients	Molecular epidemiological study	1	42	—	—
18	Çavuşoğlu and Yılmaz [30]	Turkey	TB patients	Molecular epidemiological study	13	482	European 2	MIRU‐VNTR
19	Aljameel et al. [31]	Sudan	TB patients	Molecular epidemiological study	3	179	—	Spoligotyping
20	Azami et al. [32]	Morocco	TB patients	Molecular epidemiological study	6	215	European 1	MIRU‐VNTR
21	Çakir et al. [33]	Turkey	TB patients	Molecular epidemiological study	18	412	European 2	Spoligotyping
22	Al‐Saqur et al. [34]	Iraq	TB patients	Molecular epidemiological study	2	73	—	Spoligotyping
23	Elsayed et al. [35]	Egypt	TB patients	Molecular epidemiological study	1	67	—	RFLP
24	El‐Seedy et al. [36]	Egypt	TB patients	Molecular epidemiological study	1	45	—	—
25	Tadayon et al. [37]	Iran	TB patients	Molecular epidemiological study	14	1328	—	—
26	Hamid et al. [38]	Morocco	Abattoir workers	Cross‐sectional molecular study	6	92	—	—
27	Abdel‐Moein et al. [39]	Egypt	Farmers/livestock handlers	Cross‐sectional molecular study	5	88	—	—

Each study was categorized as having low, moderate, or high risk of bias depending on the number of criteria fulfilled. This quality assessment allowed for the interpretation of findings in relation to methodological robustness and helped identify potential limitations in the evidence base [40].

### 2.7. Statistical Analysis

Statistical analyses were conducted using R software (version 4.5.3) with the *meta* and *metafor* packages. A random‐effects model was used to estimate the pooled prevalence of human *M. bovis* infection with the corresponding 95% confidence intervals. Random‐effects modeling was selected because differences in populations, study settings, diagnostic approaches, and study designs were expected to contribute to between‐study variability [41].

Heterogeneity among studies was assessed using the *I* ^2^ statistic, which quantifies the proportion of total variation due to heterogeneity rather than chance. Values of 25%, 50%, and 75% were interpreted as low, moderate, and high heterogeneity, respectively. Subgroup analysis by occupational or other predefined risk groups was planned a priori; however, insufficiently reported and noncomparable data prevented meaningful quantitative subgroup analysis. This limitation was considered in the interpretation of the findings. Sensitivity analyses were also conducted by sequentially removing individual studies to determine the stability and robustness of pooled estimates. These analyses ensured that no single study disproportionately influenced the overall results.

Publication bias was assessed using funnel plot visualization and Egger’s regression test. Funnel plot asymmetry was used to detect potential small‐study effects, while Egger’s test provided a statistical evaluation of bias.

## 3. Result

### 3.1. Study Characteristics

The literature search identified a total of 1555 records from five electronic databases and manual reference–list screening: PubMed/MEDLINE (*n* = 360), Scopus (*n* = 330), Web of Science (*n* = 280), Embase (*n* = 310), Google Scholar (*n* = 207), and manual reference–list screening (*n* = 68). After the removal of duplicate records and other ineligible records, the remaining records underwent title and abstract screening, followed by full‐text assessment against the predefined eligibility criteria. Ultimately, 27 studies met the inclusion criteria and were included in the systematic review and quantitative synthesis. The complete study selection process is presented in Figure 1. The selection procedure of the literature was based on PRISMA guidelines, and it is shown by Figure 1.

Most of the included studies were conducted among individuals diagnosed with TB who passively attended health service facilities for diagnosis and treatment. The number of confirmed cases varied considerably across studies, ranging from a single case in some reports to more than 150 cases in others. Although occupationally exposed populations were considered in the predefined review scope, occupational status and other risk‐group characteristics were inconsistently reported across studies. The available information was therefore insufficient and insufficiently comparable to support a formal subgroup meta‐analysis by occupational exposure.

Molecular characterization of isolates was reported in several studies. A variety of spoligotype patterns were detected, including SB0120, SB0121, SB0134, SB0288, SB0140, SB0989, SB2025, SB1200, and SB1003. These patterns were occasionally linked to specific clonal complexes, most commonly the Eu2 clonal complex, while other clonal complexes such as Eu1 were also identified (Table 1).

A range of genotyping techniques was applied to studies to determine strain diversity and transmission patterns. Frequently used approaches included spoligotyping combined with MIRU‐VNTR, while other studies used whole genome sequencing, PCR‐based methods, IS6110‐RFLP, or GenoType MTBC assays. However, several studies did not report detailed molecular typing results.

### 3.2. Burden of Human *M. bovis* Infection Reported From Individual MENA Countries

Figure 2 shows the reported prevalence of human *M. bovis* infection across countries in the MENA region. Considerable variation was observed between countries, with higher reported prevalence estimates in some settings and limited or unavailable data in others. Countries without eligible human *M. bovis* prevalence data are indicated separately in Figure 2. These differences should be interpreted cautiously because the available studies varied in population characteristics, sample size, diagnostic methods, and study design. The different sizes of the circles presented in the countries in Figure 2 show the different prevalence values. Larger blue circles show relatively higher prevalence, while smaller blue circles show lower prevalence values, and red circles show countries with no human *M. bovis* data. Countries with relatively high prevalence levels (>3%) include Tunisia, Algeria, Morocco, Egypt, and Lebanon. Countries with prevalence ranging from 2% to 3% include Iran and Iraq. While countries with <2% prevalence include Saudi Arabia, Turkey, and Sudan. On the other hand, several countries in the region lacked available data on human *M. bovis* prevalence and are represented by red circles. The presence of numerous red markers across the Gulf region and parts of the Levant indicates considerable gaps in epidemiological reporting or surveillance data.

**Figure 2:**
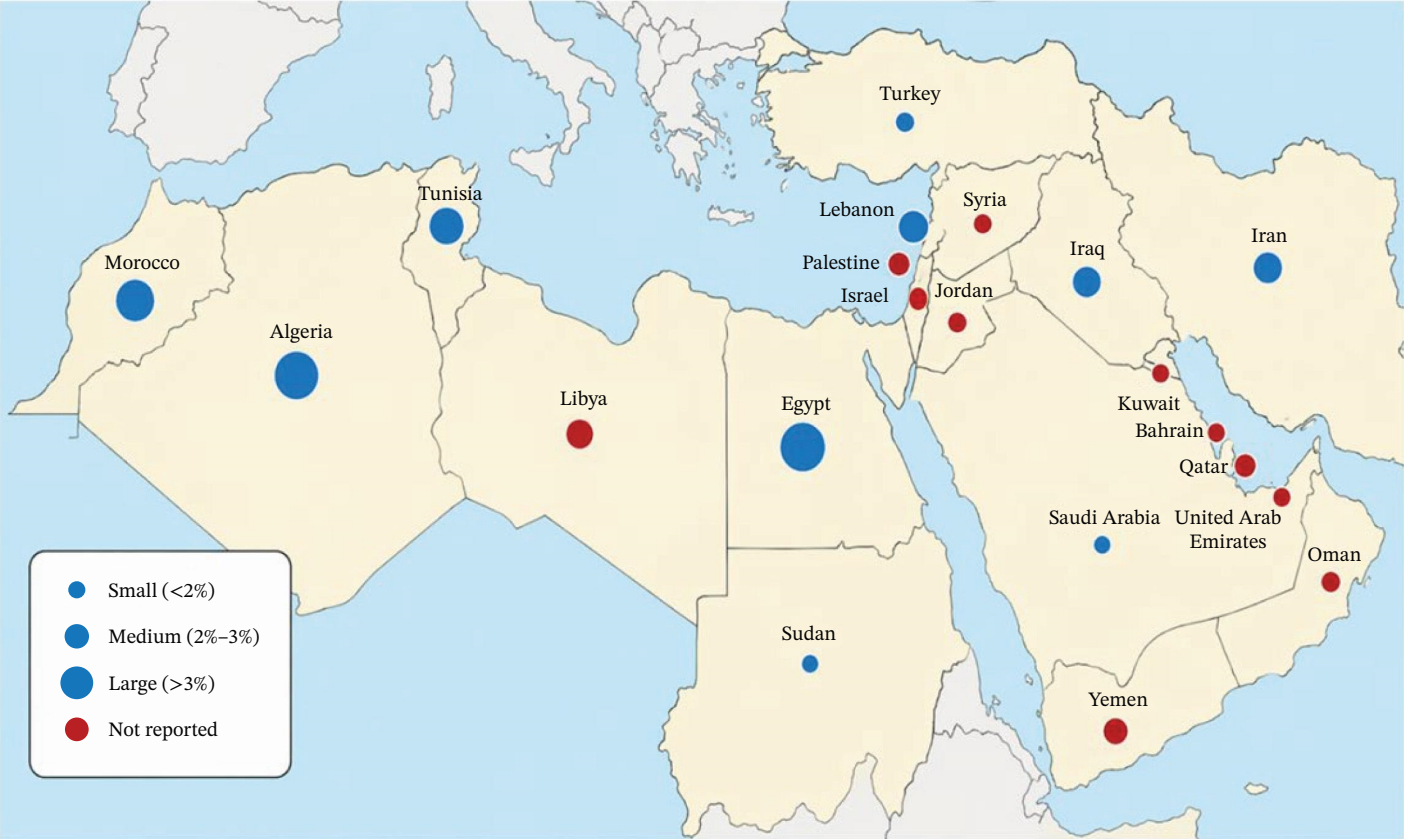
Reported prevalence of human *M. bovis* infection in MENA countries. Blue circles indicate countries with available human *M. bovis* prevalence data, whereas red circles indicate countries for which eligible prevalence data were not identified. Circle size represents the reported prevalence.

### 3.3. *M. bovis* Clonal Complexes Identified in Humans in the MENA Region

Figure 3 summarizes the reported distribution of *M. bovis* clonal complexes identified in human isolates in the MENA region. The available molecular evidence identified Eu1 and Eu2 clonal complexes. Eu2 was reported in Algeria, Tunisia, and Türkiye, whereas Eu1 was reported in Egypt and Morocco. However, molecular data were unavailable or insufficient for several countries, including Sudan, Libya, Yemen, Saudi Arabia, Jordan, Iran, Iraq, Qatar, Kuwait, Oman, Bahrain, the United Arab Emirates, Syria, Lebanon, Palestine, and Israel. Therefore, the apparent geographic distribution should not be interpreted as evidence that other clonal complexes are absent from countries without molecular surveillance data.

**Figure 3:**
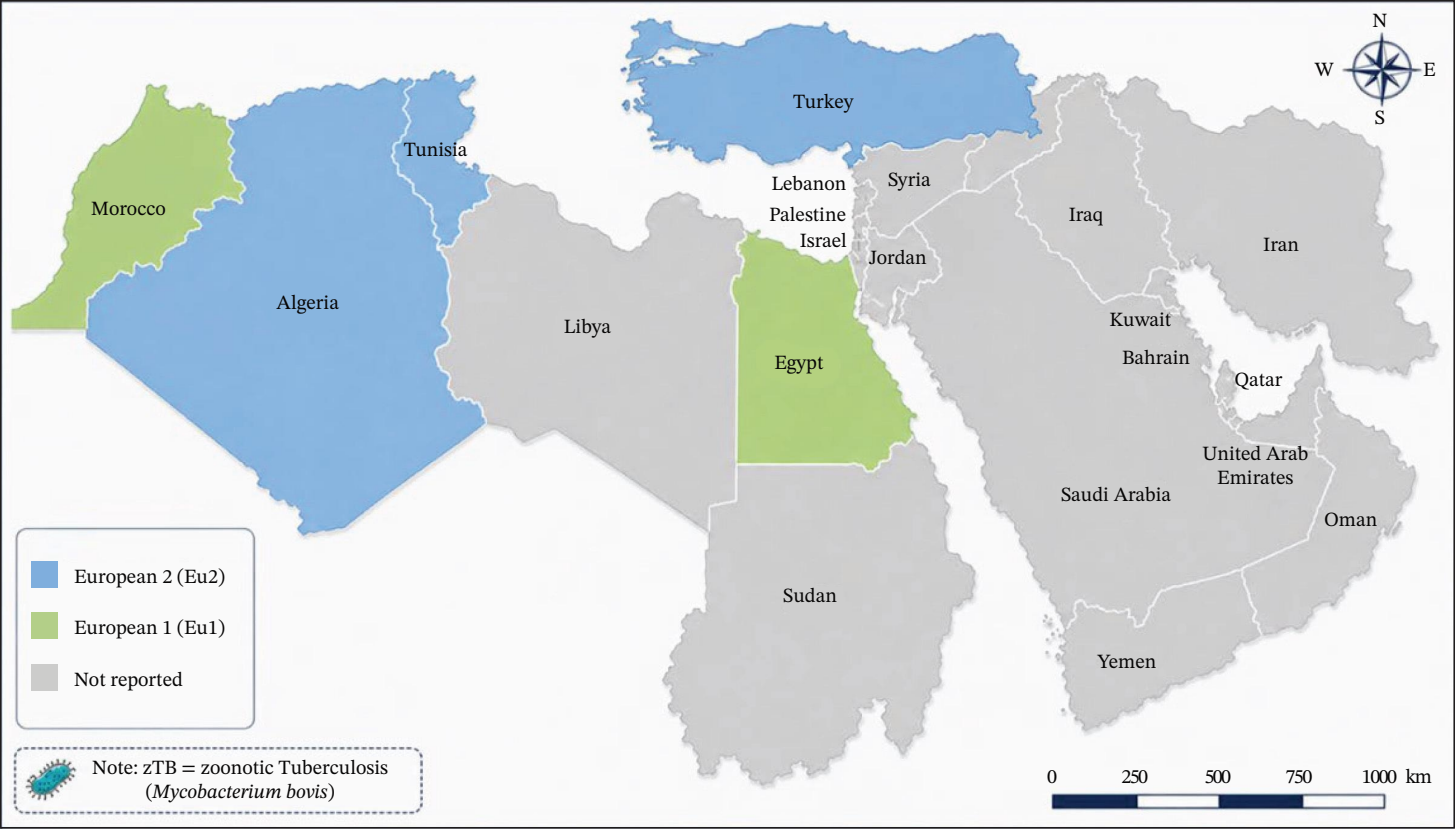
Geographic distribution of *M. bovis* clonal complexes identified in human isolates in the MENA region. European 1 (Eu1) and European 2 (Eu2) indicate clonal complexes reported among human *M. bovis* isolates. Gray‐shaded countries represent locations for which eligible human molecular data were not identified.

### 3.4. Meta‐Analysis of Human *M. bovis* Infection Prevalence

Twenty‐seven studies involving 6224 participants and 154 reported *M. bovis*–associated cases were included in the quantitative synthesis. The random‐effects model estimated a pooled prevalence of human *M. bovis* infection was 3% (95% CI: 2%–4%). Substantial between‐study heterogeneity was observed (*I* ^2^ = 73.9%, *τ* ^2^ = 0.0034, *p*  < 0.0001). The forest plot demonstrates that the confidence intervals of individual studies vary in width, largely reflecting differences in the sample size and the precision of each estimate. Larger studies generally contributed greater weight to the pooled analysis (Figure 4).

**Figure 4:**
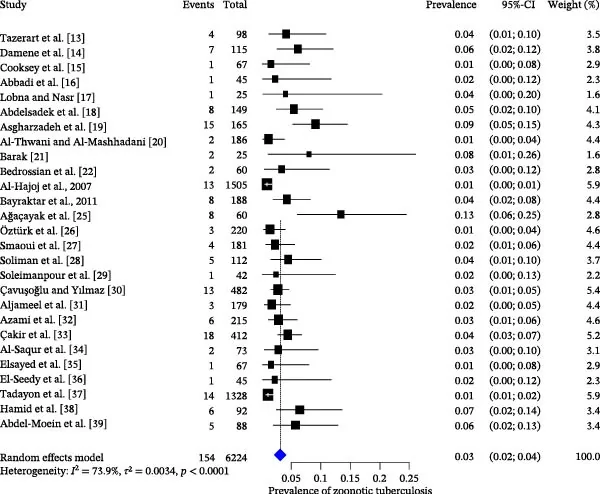
Forest plot showing the pooled prevalence of human *M. bovis* infection in the MENA region. Squares represent individual study estimates and their 95% confidence intervals, with square size proportional to study weight. The diamond represents the pooled random‐effects estimate.

#### 3.4.1. Geographic Subgroup Analysis of Pooled Prevalence of Human *M. bovis* Infection

The pooled prevalence of human *M. bovis* infection was 4% (95% CI: 3%–5%) in North Africa and 3% (95% CI: 2%–5%) in the Middle East. Heterogeneity was low in North Africa (*I* ^2^ = 0.0%, *p* = 0.5636) but substantial in the Middle East (*I* ^2^ = 85.5%, *p*  < 0.0001). The difference between the two geographic subgroups was not statistically significant (*χ* ^2^ = 0.68, df = 1, *p* = 0.408) (Figure 5).

**Figure 5:**
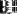
Geographic subgroup analysis of the pooled prevalence of human *M. bovis* infection in North Africa and the Middle East. This forest plot presents a subgroup meta‐analysis comparing prevalence estimates across two geographic regions: MENA. Each study is represented by a square (proportion estimate) and a horizontal line (95% confidence interval), while the pooled estimates for each subgroup and overall analysis are illustrated using diamond shapes.

#### 3.4.2. Sensitivity Analysis

The leave‐one‐out sensitivity analysis showed that the pooled prevalence of human *M. bovis* infection remained ~3% after sequential exclusion of individual studies. The corresponding 95% confidence intervals generally remained within ~2%–5%, while *I* ^2^ values ranged from ~64.5% to 74.3%. These findings indicate that the overall pooled estimate was not substantially altered by removal of any single study (Figure 6).

**Figure 6:**
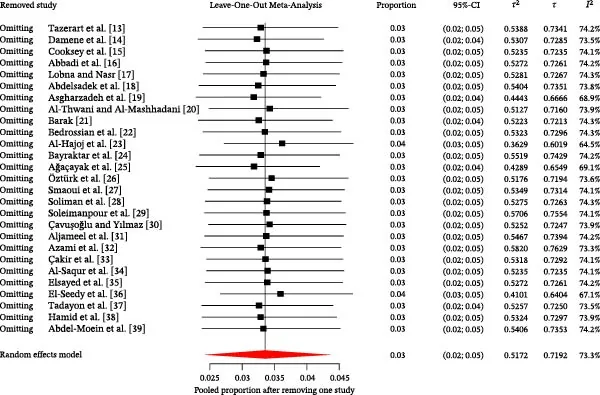
Leave‐one‐out sensitivity analysis of the pooled prevalence of human *M. bovis* infection in the MENA region. The figure presents a leave‐one‐out sensitivity analysis assessing the influence of each individual study on the overall pooled prevalence value. Each row shows the recalculated pooled estimate after removing one study at a time, along with corresponding 95% confidence intervals and heterogeneity statistics (Tau^2^, Tau, and *I* ^2^). The random‐effects model was applied throughout.

#### 3.4.3. Influence Diagnostics for Sensitivity Analysis

Influence diagnostics indicated that most studies had limited individual influence on the pooled estimate. One study showed relatively greater influence across several diagnostic measures, including studentized residuals, DFFITS, and Cook’s distance, and its removal reduced between‐study heterogeneity. Nevertheless, the leave‐one‐out analysis indicated that exclusion of individual studies did not materially change the pooled prevalence estimate. These findings suggest that the overall estimate was reasonably robust, although heterogeneity remained substantial (Figures 7 and 8).

**Figure 7:**
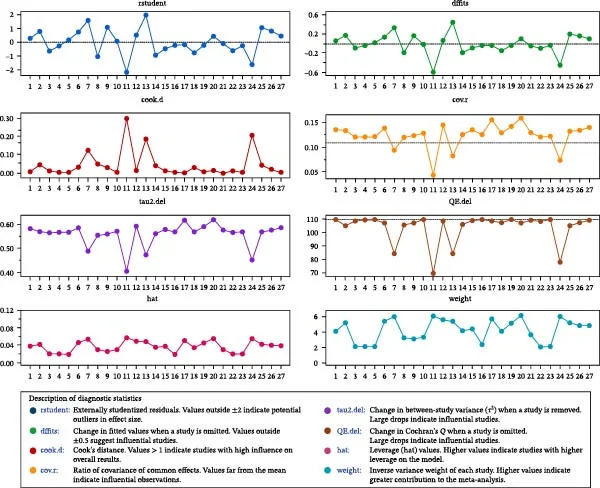
Influence diagnostics assessing the contribution of individual studies to the meta‐analysis of human *M. bovis* infection prevalence. This figure presents a comprehensive set of influence diagnostics for the meta‐analysis, including studentized residuals (rstudent), DFFITS, Cook’s distance, covariance ratios (cov.r), leave‐one‐out heterogeneity estimates (tau^2^.del), *Q*‐statistic changes (QE.del), leverage (hat values), and study weights across 27 included studies. The plots are used to assess the impact of each individual study on the pooled effect size and overall model stability. The x‐axis represents the study identification number (1 to 27). The dotted horizontal line represents the reference line (zero or mean value).

**Figure 8:**
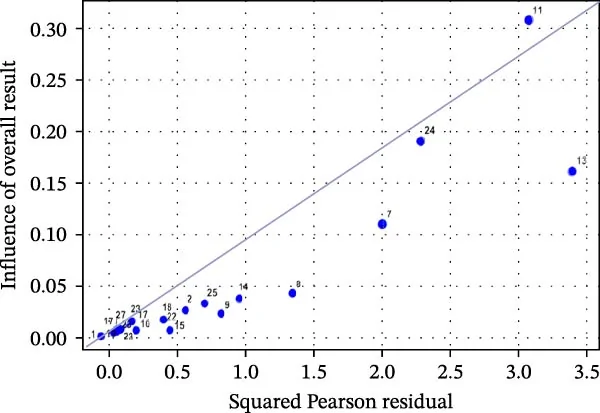
Influence plot of included studies based on squared Pearson residuals in the meta‐analysis of human *M. bovis* infection prevalence. This scatter plot displays the influence of each included study on the overall meta‐analysis estimate relative to its squared Pearson residual. Each point represents an individual study, with labels indicating study numbers. Larger residual values reflect greater deviation from the model, while higher positions on the *y*‐axis indicate stronger influence on the pooled result.

#### 3.4.4. Assessment of Publication Bias

Figure 9 shows the funnel plot that presents the result of the assessment of publication bias of the studies included in the review. The funnel plot showed some visual asymmetry, with several smaller studies distributed unevenly around the pooled estimate (Figure 9). However, visual funnel–plot interpretation is limited in the presence of substantial between‐study heterogeneity. Therefore, the observed asymmetry should not be interpreted as definitive evidence of publication bias.

**Figure 9:**
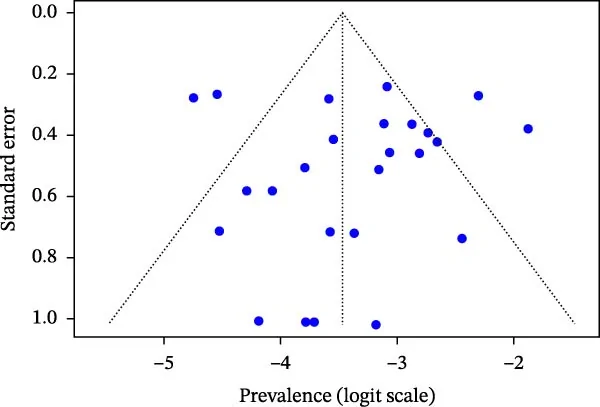
Funnel plot assessing potential small‐study effects in the meta‐analysis of human *M. bovis* infection prevalence. The *x*‐axis shows the logit‐transformed prevalence, and the *y*‐axis represents the standard error of each study. The dots indicate individual studies, the dashed vertical line shows the pooled estimate, and the dotted lines represent the pseudo 95% confidence limits. Symmetry around the pooled estimate suggests absence of bias.

#### 3.4.5. Egger’s Regression Test

Egger’s regression test produced an intercept of −0.22 (*p* = 0.852), providing no statistical evidence of small‐study effects. However, this result should be interpreted cautiously because statistical tests for funnel‐plot asymmetry may have limited power, particularly when the number of studies is modest and substantial heterogeneity is present.

The plot demonstrates a generally linear relationship between effect size and precision, with studies distributed close to the regression line (Figure 10). There is no clear visual evidence of substantial asymmetry or deviation from linearity. However, the small number of included studies limits the reliability of visual interpretation. Overall, the findings suggest no strong indication of publication bias, although this conclusion should be interpreted with caution due to the limited sample size.

**Figure 10:**
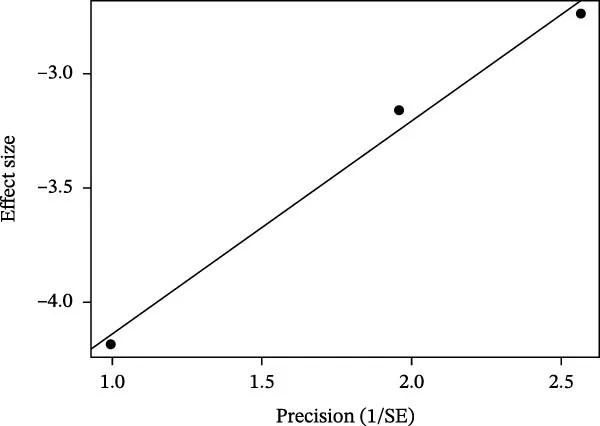
Egger’s regression plot for assessment of small‐study effects in the meta‐analysis of human *M. bovis* infection prevalence. This figure presents a regression plot of effect size against study precision (1/standard error). Each point represents an individual study included in the meta‐analysis, while the fitted regression line illustrates the relationship used to evaluate small‐study effects and potential publication bias.

## 4. Discussion

This systematic review and meta‐analysis synthesized the available evidence on human *M. bovis* infection and its molecular characteristics in the MENA region. The pooled prevalence of human *M. bovis* infection was ~3%, although substantial heterogeneity was observed among the included studies. This estimate should not be interpreted as the overall prevalence of zTB caused by all zoonotic *Mycobacterium* species because the present review specifically focused on human *M. bovis* [2, 7, 8].

The World Health Organization and multiple epidemiological reviews have consistently highlighted that the lack of species‐level identification within the MTBC leads to systematic underestimation of human *M. bovis* infection globally [2]. Consistent with this, Luciano and Roess [42] demonstrated that limited laboratory capacity and restricted access to molecular diagnostics in low‐ and middle‐income countries significantly hinder human *M. bovis* detection, particularly among populations with frequent livestock exposure.

Extending this evidence, Fareed et al. [43], in a One Health–focused review from Pakistan, reported that bTB remains endemic in livestock populations and that human *M. bovis* infections are substantially underreported due to fragmented surveillance systems. The same authors further noted that most human TB cases in Pakistan were not routinely subjected to species‐level differentiation, hence masking human *M. bovis* transmission. Furthermore, occupational exposure and consumption of unpasteurized dairy products were identified to be the key risk factors to human *M. bovis* infection.

Historically, human *M. bovis* infection has been recognized as a neglected but important component of the global TB burden [9]. Early evidence indicates that transmission from infected cattle to humans is strongly associated with inadequate bTB control measures, lack of milk pasteurization, and weak surveillance systems [6, 44]. In many low‐ and middle‐income countries, large proportions of cattle populations serve as reservoirs of *M. bovis*.

The pooled prevalence observed in the MENA region is lower than estimates reported in sub‐Saharan Africa, where studies from Ethiopia, Nigeria, and Tanzania indicate that 3%–10% of human TB cases are attributable to *M. bovis* [45, 46]. Daborn et al. [47] further demonstrated a strong association between human *M. bovis* infection in Tanzania, livestock exposure, and extrapulmonary disease. More recent studies highlight that human *M. bovis* infection remains underdetected in sub‐Saharan Africa due to weak surveillance systems and limited diagnostic capacity [48]. Similar challenges have been reported in South Asia, where fragmented surveillance and limited One Health implementation hinder accurate estimation of human *M. bovis* infection [43].

Evidence from South America suggests a comparatively lower human *M. bovis* infection burden, with sporadic cases reported mainly among rural and occupationally exposed populations [49, 50]. Similarly, in Asia, including India and neighboring countries, human *M. bovis* infection is recognized, but it is substantially underdiagnosed due to limited laboratory capacity [42, 43, 51, 52].

In contrast, Europe and North America report very low prevalence levels, generally below 1%, largely due to long‐standing bTB control programs and mandatory milk pasteurization [10, 53, 54]. Nonetheless, complete elimination has not been achieved. Roy et al. [55] reported persistent *M. bovis* cases in Spain (0.092 per 100,000 population), with spatial clustering and associations with extrapulmonary disease, indicating ongoing low‐level transmission despite strong control measures.

Occupational exposure is an important consideration in the epidemiology of human *M*. *bovis* infection because direct contact with infected livestock, animal products, and contaminated farm environments can increase opportunities for transmission [3, 7, 56]. However, the present review did not perform a quantitative comparison between occupationally exposed and nonexposed populations because occupational characteristics and exposure definitions varied substantially among studies and were not sufficiently comparable for pooled analysis. Accordingly, the available evidence should not be interpreted as demonstrating a pooled difference in *M. bovis* infection prevalence according to occupational status.

Individual studies nevertheless suggest a potential association between livestock‐related occupations and *M. bovis* infection. Fareed et al. [43] identified occupational exposure as an important predictor of infection in rural dairy settings in Pakistan, while studies from Africa have reported increased infection among populations with frequent livestock contact, particularly where consumption of raw milk is common [46, 48]. In Ethiopia, Ayalew et al. [46] reported *M. bovis* in 2.4% of culture‐confirmed TB cases and identified associations with occupational exposure and consumption of unpasteurized dairy products. Similarly, Cosivi et al. [6] highlighted raw milk consumption as an important potential route of *M. bovis* transmission in endemic settings.

Taken together, these findings support the importance of reducing exposure among populations with frequent livestock contact and consumption of unpasteurized animal products. Appropriate measures include occupational safety training, use of relevant personal protective equipment, improved food‐safety practices, targeted surveillance of high‐risk populations, and community education on the risks associated with raw milk consumption and close contact with potentially infected animals.

Animal reservoirs are central to the epidemiology of zoonotic *M*. *bovis* infection because transmission to humans can occur through direct contact with infected animals or consumption of contaminated animal products [6, 51]. Cattle and wildlife populations may therefore contribute to the maintenance and circulation of *M. bovis* in settings where livestock production and human–animal interactions are intensive. However, because the present review focused on human *M. bovis* infection, the available evidence does not allow the contribution of specific animal reservoirs to the pooled prevalence in the MENA region to be estimated directly.

Livestock production systems characterized by frequent human–animal contact and variable biosecurity practices may provide opportunities for zoonotic transmission. Nevertheless, the extent to which these factors contribute to human infection in the MENA region remains insufficiently characterized. Integrated One Health surveillance linking human, livestock, and wildlife health data would therefore be valuable for identifying animal reservoirs, clarifying transmission pathways, and improving prevention and control strategies.

Marked heterogeneity across studies reflects differences in study design, population characteristics, diagnostic approaches, and geographic coverage [41]. Consistent with this, Fareed et al. [43] reported substantial variability in estimates, largely attributable to inconsistent diagnostics and the absence of standardized surveillance systems. Diagnostic limitations remain a major barrier to accurately estimating *M. bovis* infection burden. In most MENA countries, TB diagnosis relies on smear microscopy or GeneXpert, which does not distinguish *M. bovis* from *M. tuberculosis*. Similar constraints are widely documented in other low‐ and middle‐income countries, where species‐level identification is rarely performed [42, 43, 45, 52], leading to systematic underestimation of the burden of *M. bovis* infection.

Molecular epidemiological data from the MENA region indicate that human *M. bovis* isolates predominantly belong to European clonal complexes (Eu1 and Eu2). This distribution is strongly linked to historical livestock trade and colonial‐era cattle movement between Europe and North Africa [10, 11, 57]. The available molecular evidence identified predominantly Eu1 and Eu2 clonal complexes among reported human *M. bovis* isolates in the region. Shared clonal complexes between human and animal isolates may provide evidence consistent with transmission at the human–animal interface; however, the available data are insufficient to establish specific transmission chains in most settings. Limited molecular surveillance and uneven geographic sampling also restrict conclusions regarding the true distribution of *M. bovis* clonal complexes across the MENA region.

The lack of reported Af 1 and Af 2 clonal complexes among the included human molecular studies should not be interpreted as evidence of their true absence from the MENA region. Limited sampling, incomplete molecular characterization, and uneven surveillance may contribute to the observed distribution. This interpretation is supported by the World Health Organization and Olea‐Popelka et al. [2, 8] and Fareed et al. [43], who highlight insufficient molecular epidemiological capacity as a major constraint to understanding *M. bovis* diversity and transmission dynamics.

Ecological and historical factors may also explain observed lineage distributions. Livestock trade networks in North Africa and the Middle East have historically been more closely linked to Europe and the Mediterranean region than to sub‐Saharan Africa, facilitating the introduction and persistence of European strains [10, 11]. Additionally, ecological adaptation and host population structure may influence strain establishment and persistence within specific regions [7, 58–60].

### 4.1. Public Health Implications

The findings highlight the importance of strengthening surveillance for human *M. bovis* infection as part of an integrated One Health approach. Improved species‐level diagnosis, systematic investigation of suspected zoonotic transmission, veterinary surveillance, and molecular characterization of isolates could improve the understanding of *M. bovis* epidemiology in the MENA region. Food‐safety measures, including appropriate milk pasteurization, and occupational biosafety measures may also contribute to reducing exposure where *M. bovis* infection is identified.

### 4.2. Limitations

This review has several limitations. First, substantial heterogeneity was observed among the included studies, which may reflect differences in study populations, geographical settings, study designs, sample sizes, and diagnostic approaches. Therefore, the pooled estimate should be interpreted in the context of this variability. Second, diagnostic methods differed considerably between studies, and some approaches may not reliably distinguish *M. bovis* from other MTBC members, potentially resulting in underrecognition or misclassification. Third, molecular characterization was available for only a subset of studies, limiting conclusions regarding the geographic distribution and diversity of *M. bovis* clonal complexes.

Fourth, occupational exposure and other risk‐group characteristics were inconsistently reported and could not be evaluated through a meaningful quantitative subgroup analysis. Fifth, although funnel‐plot assessment and Egger’s regression did not provide strong statistical evidence of small‐study effects, publication bias cannot be completely excluded. Sixth, the review was restricted to English‐language publications, which may have resulted in the omission of relevant evidence published in other languages. Finally, evidence was unevenly distributed geographically, with limited or no eligible human *M. bovis* data for several MENA countries. Consequently, the pooled estimate should not be generalized to countries or populations for which data were unavailable. In addition, because the review focused specifically on *M. bovis*, the findings do not represent the overall burden of zTB caused by all zoonotic *Mycobacterium* species.

## 5. Conclusion

This systematic review and meta‐analysis indicates that human *M*. *bovis* infection occurs in the MENA region, with a pooled prevalence of ~3% among the populations represented in the included studies. Considerable between‐study heterogeneity and substantial geographic and diagnostic gaps limit the interpretation and generalizability of this estimate. The available molecular evidence identified Eu1 and Eu2 clonal complexes among human *M. bovis* isolates, although molecular surveillance remains limited. These findings represent the burden of human *M. bovis* infection and should not be interpreted as an estimate of the overall burden of zTB caused by all zoonotic *Mycobacterium* species. Strengthening species‐level diagnostic capacity, molecular surveillance, and integrated One Health collaboration could improve the detection and epidemiological understanding of human *M. bovis* infection in the region.

## Author Contributions

Gobena Ameni conceived the study. Tsegaye Shamebo, Aboma Zewude, Ali Artaman, and Gobena Ameni were responsible for data curation and formal analysis. The investigation was carried out by Tsegaye Shamebo and Gobena Ameni. Methodological design was developed by Tsegaye Shamebo and Gobena Ameni. Resource and software management were handled by Tsegaye Shamebo, Ali Artaman, and Gobena Ameni. Supervision and oversight were provided by Ali Artaman and Gobena Ameni. Data validation was performed by Tsegaye Shamebo, Aboma Zewude, Temesgen Mohammed, Ali Artaman, and Gobena Ameni. Visual data presentations and figures were prepared by Tsegaye Shamebo and Gobena Ameni. The initial draft of the manuscript was written by Tsegaye Shamebo. Aboma Zewude, Temesgen Mohammed, Nabeeha Abdelgaleel Hassan, Robert Barigye, Markos Tibbo, Ali Artaman, and Gobena Ameni contributed to reviewing of the manuscript.

## Funding

The authors declared that financial support was received for this work and/or its publication. The UAE University (UAEU) and Zayed University (ZU) jointly funded this study (Grant 12R343 and Grant 23215), respectively.

## Disclosure

Aboma Zewude, Temesgen Mohammed, Nabeeha Abdelgaleel Hassan, Robert Barigye, Markos Tibbo, Ali Artaman, and Gobena Ameni approved the manuscript.

## Conflicts of Interest

The authors declare no conflicts of interest.

## Supporting Information

Additional supporting information can be found online in the Supporting Information section.

## Supporting information


**Supporting Information** Newcastle–Ottawa Scale quality assessment of the studies used for the meta‐analysis. The methodological quality and risk of bias of the studies were assessed using an adapted Newcastle–Ottawa Scale (ANOS). The assessment considered study design, sampling methodology, population representativeness, diagnostic methods, outcome ascertainment, and completeness of reporting.

## Data Availability

The data that support the findings of this study are available from the corresponding author upon reasonable request.
