# Supplementary material for: The Burden and Genetic Diversity of Mycobacterium bovis Infection in Humans in the Middle East and North Africa: A Systematic Review and Meta‐Analysis
Source: Transbound Emerg Dis. 2026 Oct 6;2026:5876735. doi: 10.1155/tbed/5876735 (PMC13639931; doi:10.1155/tbed/5876735)
Supplement: Supplementary file 1 [file TBED-2026-5876735-s001.docx]

**Supplementary S1. Newcastle–Ottawa Scale Quality Assessment of the studies used for the meta-analysis**

| **Study No.** | **Author** | **Selection (0–4)** | **Comparability (0–2)** | **Outcome (0–3)** | **Total (0–9)** | **Quality** |
| --- | --- | --- | --- | --- | --- | --- |
| 1 | Tazerart et al., 2021 | 4 | 1 | 3 | 8 | High |
| 2 | Damene et al., 2020 | 4 | 1 | 3 | 8 | High |
| 3 | Cooksey et al., 2020 | 3 | 1 | 2 | 6 | Moderate |
| 4 | Abbadi et al., 2009 | 2 | 0 | 2 | 4 | Low |
| 5 | Lobna & Nasr, 2015 | 2 | 0 | 1 | 3 | Low |
| 6 | Abdelsadek et al., 2020 | 4 | 1 | 2 | 7 | High |
| 7 | Asgharzadeh et al., 2011 | 4 | 2 | 3 | 9 | High |
| 8 | Al-Thwani & Al-Mashhadani, 2016 | 2 | 0 | 1 | 3 | Low |
| 9 | Barak, 2012 | 2 | 0 | 1 | 3 | Low |
| 10 | Bedrossian et al., 2013 | 3 | 1 | 2 | 6 | Moderate |
| 11 | Al-Hajoj et al., 2007 | 4 | 2 | 3 | 9 | High |
| 12 | Bayraktar et al., 2011 | 3 | 1 | 2 | 6 | Moderate |
| 13 | Ağaçayak et al., 2007 | 2 | 0 | 1 | 3 | Low |
| 14 | Öztürk et al., 2016 | 3 | 1 | 2 | 6 | Moderate |
| 15 | Smaoui et al., 2015 | 3 | 1 | 2 | 6 | Moderate |
| 16 | Soliman et al., 2024 | 4 | 2 | 3 | 9 | High |
| 17 | Soleimanpour et al., 2015 | 2 | 0 | 1 | 3 | Low |
| 18 | Cavusoglu et al., 2017 | 4 | 2 | 3 | 9 | High |
| 19 | Musa et al., 2015 | 3 | 1 | 2 | 6 | Moderate |
| 20 | Bouslikhane et al., 2020 | 4 | 2 | 3 | 9 | High |
| 21 | Yildirim et al., 2018 | 4 | 2 | 3 | 9 | High |
| 22 | Al-Hamdani et al., 2016 | 2 | 0 | 1 | 3 | Low |
| 23 | El-Sayed et al., 2000 | 2 | 0 | 1 | 3 | Low |
| 24 | Hassan et al., 2007 | 2 | 0 | 1 | 3 | Low |
| 25 | Oryan et al., 2008 | 3 | 1 | 2 | 6 | Moderate |
| 26 | Benjelloun et al., 2019 | 3 | 1 | 2 | 6 | Moderate |
| 27 | Khalil et al., 2017 | 3 | 1 | 2 | 6 | Moderate |
